# Functional significance of the hepaCAM gene in bladder cancer

**DOI:** 10.1186/1471-2407-10-83

**Published:** 2010-03-08

**Authors:** Yunfeng He, Xiaohou Wu, Chunli Luo, Lie Wang, Jie Lin

**Affiliations:** 1 Department of Urology, The first affiliated hospital, ChongQing Medical University, 1 YouYi Road, Central District, ChongQing 400016, China; 2 Faculty of laboratory medicine of ChongQing Medical University, 1 YouYi Road, Central District, ChongQing 400016, China

## Abstract

**Background:**

The hepaCAM gene encodes a new immunoglobulin-like cell adhesion molecule, and its expression is suppressed in a variety of human cancers. Additionally, hepaCAM possesses properties often observed in tumor suppressor genes. However, the expression and biological function of hepaCAM has not been investigated in bladder cancer. Therefore we sought to examine hepaCAM expression and the relationship between its structure and function in human transitional cell carcinoma of bladder (TCCB).

**Materials and methods:**

HepaCAM expression was evaluated in 28 normal and 34 TCCB bladder specimens and 2 TCCB cell lines using semi-quantitative RT-PCR. The wild-type hepaCAM and the extracellular domain-truncated mutant gene were transfected into the TCCB cell line T24, and the biological properties of both the wild-type gene and the domain-truncated mutant were then assessed.

**Results:**

HepaCAM expression was down-regulated in 82% (28/34) of TCCB specimens and undetectable in the 2 TCCB cell lines tested. The localization of hepaCAM appeared to be dependent on cell density in T24 cells. In widely spread cells, hepaCAM accumulated on the perinuclear membrane and the cell surface protrusions, whereas in confluent cells, hepaCAM was predominantly localized at the sites of cell-cell contacts on the cell membrane. Functionally, hepaCAM expressed not only increased cell spreading, delayed cell detachment, enhanced wound healing and increased cell invasion; it also inhibited cell growth (P < 0.01). When the extracellular domain was deleted, the localization of hepaCAM was significantly altered, and it lost both its adhesive function and its influence on cell growth.

**Conclusions:**

HepaCAM is involved in cell adhesion and growth control, and its expression is frequently silenced in TCCB. The extracellular domain of hepaCAM is essential to its physiological and biological functions.

## Background

Cell-cell and cell-matrix adhesion is essential for normal organogenesis and for the maintenance of normal tissues in humans. This adhesion is largely mediated by a large and complex number of adhesion molecules expressed on the cell surface [1]. They are generally classified into four types based on their structural and functional features: cadherins, selectins, integrins, and members of the immunoglobulin superfamily (IgSF) [2-5]. An exciting concept that has recently emerged from recent cell biological research is that cell adhesion complexes are not simply static architectural entities that play a role in cell-cell and cell-matrix adhesion. Rather, these complexes are dynamic units that play a critical role in modulating cytoplasmic signaling cascades by capturing and integrating signals from the extracellular environment [6]. These interactions are vital for the regulation of cellular adhesion, proliferation, apoptosis, migration, and differentiation.

Recently, Mei Chung Moh et al. identified a novel gene in the liver that they called hepaCAM (hepatocyte cell adhesion molecule); this gene is differentially expressed in human hepatocellular carcinoma as compared to normal liver tissue [7,8]. hepaCAM is located on chromosome 11q24, contains 7 exons, and encodes a novel 416 amino acid protein. The protein possesses a structure that is typical of Ig-like adhesion molecules, including two extracellular Ig-like domains, a transmembrane segment and a cytoplasmic tail [9,10]. Previous studies have shown that the expression of hepaCAM is frequently completely suppressed in human hepatocellular carcinoma and significantly suppressed in a variety of other tumor types including malignancies of the lung, brain, colon, and blood. When transfected into human hepatocellular carcinoma HepG2 and breast carcinoma MCF7 cells, the wild-type hepaCAM not only increases cell spreading on Matrigel matrices, delays cell detachment, and enhances wound healing, but it also reduces colony formation and inhibits cell growth. It was recently found that deletion of the cytoplasmic domain of hepaCAM did not interfere with dimerization, but that its presence was necessary for hepaCAM to exert its normal physiological and biological functions [7,9,10]. Another study showed that deletion of either the extracellular or the cytoplasmic domain of hepaCAM abolished actin co-precipitation and delayed cell-ECM adhesion and cell motility [11]. Additionally, it was recently shown that the extracellular first immunoglobulin domain of hepaCAM was required for the binding of the caveolar structural protein caveolin-1 [12]. However, the structural domains necessary for the normal function of hepaCAM remain uncharacterized in the renal system.

Therefore, in this study, we sought to investigate the expression of hepaCAM in TCCB as well as the physiological and biological properties of hepaCAM in the TCCB cell line T24. We showed that hepaCAM is expressed in normal bladder tissues but that its expression is decreased or absent in both the TCCB samples we included in our study and the TCCB cell lines T24 and BIU-87. When transfected into T24 cells, hepaCAM not only increases cell spreading on Matrigel matrices, delays cell detachment, and enhances wound healing, but it also inhibits cell growth. Interestingly, a hepaCAM mutant with truncated extracellular domain no longer exhibits these biological characteristics in T24, indicating the importance of the extracellular domain in the function of hepaCAM in bladder tissue.

## Methods

### Patient characteristics

All of the tumor samples were obtained from patients newly diagnosed and treated for TCCB at our department between 2005 and June 2007. Clinical information regarding these patients was retrieved from medical records. Carcinoma specimens were obtained from 34 patients (28 men and 6 women, age range 38 to 76 years) with primary or recurrent bladder cancer. All of the tumors were initially diagnosed as bladder carcinoma before surgery based on clinical examinations including urinary cytology and cystoscopy. Histological examination of the tumor tissues after surgery confirmed that all patients had transitional cell carcinoma. According to the guidelines of the International Union Against Cancer, the histologic grade and stage were determined as follows: Grade G1 in 16; G2 in 8; and G3 in 10; and Stage Ta-T1 in 10; T2-T4 in 24. Normal bladder tissue samples were surgically excised from 28 unpaired patients (24 men and 4 women, age range 46 to 69 years) without cancer and were confirmed as normal by histological examination. Patients with bladder cancer received standardized treatments and underwent identical post-treatment surveillance. The institutional review board approved the study protocol. All patients provided informed consent for the use of their bladder specimens.

### Cell lines

Two human TCCB cell lines, T24 and BIU-87, were provided by the Basic Medical Sciences Laboratory of Chongqing Medicine University and maintained and grown in RPMI-1640 medium supplemented with 10% fetal bovine serum, 50 U/mL penicillin, and 50 μg/mL streptomycin in a 5% carbon-dioxide-containing humidified incubator at 37°C.

### RT-PCR and determination of relative hepaCAM expression levels

The primers and probes for the hepaCAM gene were chosen with the assistance of the computer program Primer Premier 5.0 (PremierBiosoft, USA). We then conducted a BLASTN search using the GenBank database to confirm the total gene specificity. A forward primer (5'-TAC TGT AGA TGT GCC CAT TTC G-3) and reverse primer (5'-CTT CTG GTT TCA GGC GGT C-3') were used to generate a hepaCAM fragment of 461 bp from 0.1 μg DNase-treated total RNA. Beta-actin was used as a control. Semi-quantitative RT-PCR reactions were performed with the Two-step RT-PCR kit (Takara, Japan) following the manufacturer's protocol. Polymerase chain reaction (PCR) was carried out using the primers for the indicated genes under optimized conditions. Details regarding the PCR conditions and gene-specific primers are available on request.

The semi-quantitative analysis of the amplified PCR products was performed using a BIO-RAD imaging plate (BIO-RAD, USA). All samples were analyzed simultaneously. The relative amount of each hepaCAM mRNA was normalized to a beta-actin signal from the same sample. The results are expressed as the hepaCAM/beta-actin ratio, which was defined as the expression level for the respective hepaCAM gene.

### Biological properties of the wild-type and mutant hepaCAM genes in T24 cells

The plasmids containing wild type hepaCAM and the mutant hepaCAM with a truncated extracellular domain were provided by Dr. Shali Shen, and the sequences of the recombinant plasmids were verified by sequencing. The complete coding sequences of hepaCAM and its truncated extracellular domain mutant were generated by PCR amplification. The cDNAs of wild-type hepaCAM (residues 1-416) and mutant hepaCAM (residues 240-416) were cloned into the pEGFP-N2 vector (Clontech, Palo Alto, CA) at the HindIII/BamHI restriction sites. The constructs were named hepaCAM-GFP and hepaCAM-mt1-GFP, respectively. When the transfected T24 cells expressed hepaCAM-GFP or hepaCAM-mt1-GFP, this could be detected by an anti-GFP antibody, fluorescence microscopy, or confocal microscopy. Transient transfections were carried out with Lipofectamine 2000TM (Invitrogen). T24 cells grown on coverslips were transfected with hepaCAM-GFP, hepaCAM-mt1-GFP, and pEGFP-N2 vector. Then, these cells were selected using 400 μg/ml of G418 for 4 weeks, and subsequently cloned.

Total protein (50 μg) from the cells was resolved by SDS-PAGE, transblotted onto a membrane (Santa Cruz, CA), and detected using a rabbit anti-GFP monoclonal antibody (Santa Cruz, CA). The membranes were then stripped with a mouse anti-rabbit antibody to assess loading quantity.

Cells were seeded in plates coated with 40 μg of Matrigel basement membrane matrix (Collaborative Research) and incubated under standard conditions. Cell morphology was observed by microscopy. Unspread cells were defined as round cells, while spread cells were defined as cells with extended cellular processes. The percentage of cells demonstrating a spread morphology was quantified in 10 randomly selected fields (>60 cells/field).

A confluent monolayer of cells was detached using 1 mM EDTA in PBS at 37°C. Cell detachment was evaluated under the inverted microscope after 5 minutes and 15 minutes of incubation. Concurrently, the dissociated cells were harvested and counted in 10 randomly selected fields.

Cells were seeded onto 35-mm culture plates at high density and allowed to form monolayers overnight. Then, the cells were wounded with a sterile 200-μl plastic micropipette tip. The wound was examined microscopically at 12 and 24 hours. The changes in diameter (D) of each wound were measured by microscopy and computed into a ratio (D24/Dinitial × 100%) reflecting the degree of wound closure that had occurred.

Cell invasion was assessed using transwell chambers with 8-μg pore-size membranes coated with Matrigel (Collaborative Research) that were placed in 24-well plates. Cells (5 × 10^4^) were loaded into the upper segment of the chambers and allowed to invade through the membrane for 24 h. In the upper segment of the chambers is the 5% fetal bovine serum, in the under chambers is the 10% fetal bovine serum, The cells will growth form low level serum to higher level serum. The invasion cells were harvested by trypsinizing the lower surface of the membrane and were collected. Non-migrated cells were then removed. The invasion cells were harvested by trypsinizing the lower surface of the membrane and were collected and seeded into a new 24-well plate, 500 μl 0.1%gentian violet was add into 24-well plate, place the membrane into it, in 37°C take out the membrane after 30 min, wash the membrane with PBS. The invasion activity was then quantified by blind counting of the invasion cells in 10 randomly selected microscopic fields (>40 cells/field).

The growth rate of T24 cell lines were monitored for seven days. Cells were seeded in triplicate and cultured under standard conditions. Every 24 h, cell viability was determined by MTT assay. The growth rate of each cell line was presented as fold of increase in cell viability as compared to the respective baseline value.

### Statistical analysis

Statistical analyses were performed using the Statistical Package for Social Sciences version 13.0, for Windows. Nonparametric ANOVA was performed to compare the difference among more than two parameters. Fisher's exact test was used to assess the correlation between two parameters. Statistical significance in this study was set at P < 0.05. All reported P values are two-sided.

## Results

### Expression of hepaCAM mRNA and its correlation with clinical and patholagical features of bladder cancer

**Figure 1:**
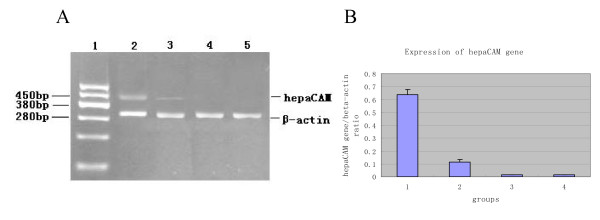
**The expression of hepaCAM gene as determined by semi-quantitative RT-PCR**. Products were analyzed on a 1.5% agarose gel. The 461-bp band is the wild-type hepaCAM gene. Line 1 is the marker. Lines 2 to 5 exhibit the expression of hepaCAM gene in normal bladder tissue specimens, TCCB specimens, T24 cells, and BIU-87 cells(A). Quantified expression levels of hepaCAM normalized to β-actin expression levels. Groups 1 to 4 exhibit hepaCAM gene expression levels in normal bladder tissue specimens, TCCB specimens, T24 cells, and BIU-87 cells(B).

Semi-quantitative RT-PCR revealed that hepaCAM was expressed at a similar level in all of the 28 normal bladder tissue specimens that we tested. We also examined hepaCAM mRNA levels in 34 TCCB specimens. The results showed that hepaCAM expression was reduced in 82% (28/34) of TCCB tissues. The expression of hepaCAM was not detectable in eight TCCB specimens and in the TCCB cell lines we tested. These data implied an association between the loss of hepaCAM expression and the presence of TCCB. However, no statistically significant difference could be detected when we examined possible correlations between hepaCAM expression levels and various clinicopathological parameters [Fig. 1A, B. Table 1].

**Table 1:** Correlation between hepaCAM suppression and clinicopathological parameters of the 34 TCCB specimens

Parameters	hepaCAM Suppression	Unchanged hepaCAM	Suppression rate (%)	*P < 0.05*
Total number	28	6	82	
*Sex*				
Male	22	6	79	NS
				
Female	6	0	100	NS
Histologic *grade*				
Ta-T1	4	6	40	
T2-T4	24	0	100	
Histologic *stage*				
G1	14	2	88	NS
G2	7	1	88	NS
G3	7	3	70	NS
*occurrence*				
Primary	16	4	80	NS
Recurrence	12	2	86	NS

KEY: NS = not significant; * Mann-Whitney U test; P < 0.05 was considered statistically significant

### The biological properties of hepaCAM in the T24 cell line

Western blot analysis (using a rabbit anti-GFP monoclonal antibody for detection) revealed the results of the transfections we performed. In the T24 cells transfected with pEGFP-N2, a specific 27-kDa band was observed. In the T24 cells transfected with hepaCAM or hepaCAM-mt1, 100 kDa or 75 kDa bands, respectively, were observed.

**Figure 2:**
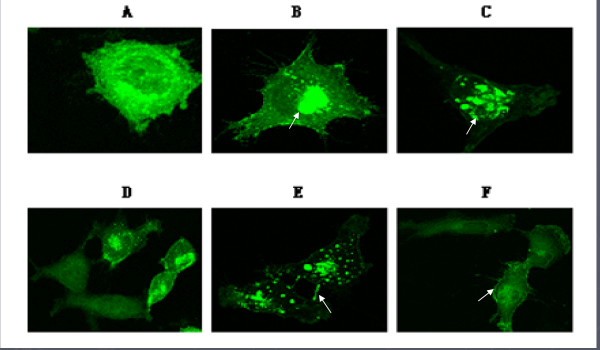
**Subcellular localization of hepaCAM and its extracellular domain truncation mutant in T24 cells**. In well-spread cells, hepaCAM and its extracellular domain truncation mutant were localized on the perinuclear membrane and at the tip of cell surface protrusions. [B, C (white arrows indicate the localization of wild-type hepaCAM and its mutant), A is the control group]. In confluent cells, hepaCAM predominantly accumulated at the sites of cell-cell contacts on the cell membrane (E). However, in confluent cells, hepaCAM-mt1 did not accumulate at the sites of cell-cell contact, but instead localized on the cell membrane non-specifically (F, white arrows indicate localization of hepaCAM-mt1). D is the control group. 1000× magnification.

To determine the localization of hepaCAM, T24 cells were transiently transfected with either hepaCAM-GFP or hepaCAM-mt1-GFP. The GFP vector alone served as a control. As visualized by confocal microscopy, in well-spread cells, hepaCAM was localized on the pronuclear membrane, in the cytoplasm, and at the tip of cell surface protrusions that were about to make contact with adjacent cell surfaces. However, in confluent cells, hepaCAM was found to be predominantly accumulated at the sites of cell-cell contacts on the cell membrane. In the T24 cells, hepaCAM-mt1 was also predominantly localized on the perinuclear membrane in the well-spread cells, but was in the confluent cells, it was non-specifically localized. These results revealed a difference in the subcellular localization of hepaCAM and hepaCAM-mutant in T24 cells, implying that the extracellular domain may participate in the subcellular localization of hepaCAM [Fig. 2].

We evaluated the adhesive effects of hepaCAM and its extracellular domain truncation mutant on T24 cells using cell adhesion and detachment assays. Our data showed that on Matrigel, T24/hepaCAM cells spread the fastest, followed by T24/hepaCAM-mt1 cells, with the T24/pEGFP-N2 cells showing the slowest rate of spread. A total of 57.4% and 94.2% of the T24/hepaCAM cells exhibited a well-spread morphology on Matrigel after 30 minutes and 2 hours of incubation, respectively. In contrast, only 7.3% and 19.1% of the T24/hepaCAM-mt1 cells and 7.3% and 21.9% of T24/pEGFP-N2 cells were found to be well-spread at the same respective times (P < 0.01). In the cell detachment assay, T24/hepaCAM cells detached 7.0 times and 10.4 times slower than T24/pEGFP-N2 cells at 5 minutes and 15 minutes, respectively. On the other hand, the detachment rate of T24/hepaCAM-mt1 cells (52.7% and 97.4%) was not statistically different than that of the control cells (T24/pEGFP-N2) [51.5% and 97.6%; (P > 0.05)].

**Figure 3:**
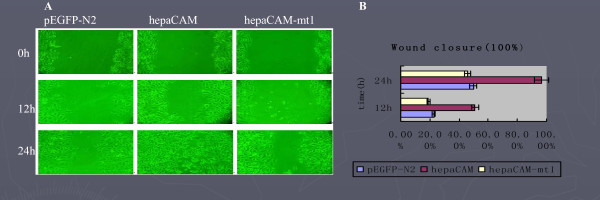
**Wound healing assay**. Wounds were made with a pipette tip on confluent T24/pEGFP-N2 (left panel), T24/hepaCAM (middle panel), and T24/hepaCAM-mt1 (right panel) cells and allowed to heal for 12 and 24 h. 100× magnification(A). The diameters of wounds were measured on the microscopic photos at 0 h, 12 h, and 24 h after the wound was induced. Changes in wound diameter were computed into percentages (means ± SD) to represent wound closure. P < 0.01 as assessed by ANOVA(B).

Cell motility mediated by hepaCAM and its extracellular domain truncation mutant was assessed by wound healing and Matrigel invasion assays. As revealed by the wound healing assay [Fig. 3], after 12 hours of incubation, 50.9% of the scratched area was filled by T24/hepaCAM cells, in contrast to 19.0% by T24/hepaCAM-mt1 cells and 21.7% by T24/pEGFP-N2 cells (P < 0.01). After 24 h of incubation, 96.6% of the scratched area was filled by T24/hepaCAM cells, 46.0% by T24/hepaCAM-mt1 cells, and 49.8% by T24/pEGFP-N2 cells (P < 0.01). In addition, the Matrigel invasion assays showed that T24/hepaCAM cells had a higher degree of motility than both T24/hepaCAM-mt1 and the control T24/pEGFP-N2 cells, The invasion ability of T24/hepaCAM group(102.30 ± 16.06 cells/field) is higher than the T24/hepaCAM-mt1 group and control T24/pEGFP-N2 group (*P *< 0.05), T24/hepaCAM-mt1 group (43.20 ± 10.45 cells/field)and control T24/pEGFP-N2 group(41.00 ± 9.94 cells/field)is no statistics difference(*P *> 0.05). It was noteworthy that both the migration and invasion capacities of the cells expressing the hepaCAM mutant were comparable to that of the control cells, indicating that the extracellular domain was essential for hepaCAM-mediated cell motility and invasion.

**Figure 4:**
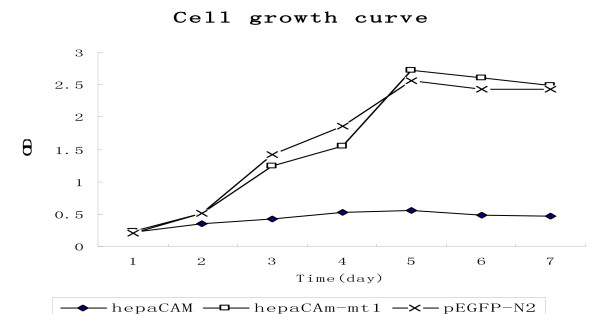
**Inhibition of cell growth by hepaCAM**. The growth rate T24/hepaCAM and T24/hepaCAM-mt1 with T24/pEGFP-N2 cells measured over a seven day period by MTT assay. Data represent means ± SD. P < 0.01 as assessed by ANOVA.

To examine the involvement of hepaCAM and its mutant in the regulation of cell growth, the cell growth rate was determined in the stable T24 cell clones. The results showed that the cell growth rate was reduced about 5-fold in the T24/hepaCAM cells as compared to the T24/pEGFP-N2 cells (P < 0.01). The growth rate of T24/hepaCAM-mt1 cells was not significantly different than that of T24/pEGFP-N2 cells [Fig. 4].

## Discussion

HepaCAM was recently demonstrated to be differentially expressed in human hepatocellular carcinoma [13,14]. Studies have shown that the expression of hepaCAM is frequently lost in human hepatocellular carcinoma and processed a tumor suppressor gene [7,15,16]. However, hepaCAM has not been previously investigated in TCCB. It is well known that the occurrence and development of human TCCB demonstrates many similarities to that of HCC. Our hypothesis is that hepaCAM may play similar roles in TCCB as in HCC. To test our hypothesis, we examined the expression of 28 normal bladder tissue specimens, 34 TCCB specimens, and 2 TCCB cell lines, T24 and BIU-87. The results showed that the level of expression of hepaCAM in all of 14 normal bladder tissue specimens was similar, but that it was decreased in 85% (28/34) of TCCB specimens. Moreover, the expression of hepaCAM was absent in 26% (8/34) of TCCB specimens and both TCCB cell lines.

To further evaluate whether or not hepaCAM possessed any functional roles in the bladder, we assessed the physiological and biological characteristics of hepaCAM in the TCCB cell line T24. Investigations were carried out to reveal the relationship between the structure and function of hepaCAM, especially with respect to the extracellular domain in the bladder. We constructed two eukaryotic expression vectors containing either the wild-type or the extracellular domain-truncated mutant of hepaCAM. The subcellular localization of hepaCAM appears to depend on cell density in T24 cells. When transfected into T24 cells, hepaCAM is localized on the perinuclear membrane, in the cytoplasm, and at the tip of cell surface protrusions that are about to make contact with adjacent cell surfaces in well-spread cells, whereas in confluent cells, hepaCAM is found to be predominantly accumulated at the sites of cell-cell contacts on the cell membrane. This finding is similar to that reported in HepG2 cells, an HCC cell line. Through cell adhesion and motility assays, we have observed that hepaCAM increases cell spreading on Matrigel, delays cell detachment, enhances wound healing and increases cell invasiveness. The reason that hepaCAM may still be a tumor suppressor despite the fact that it increases cell invasiveness is due to the fact that hepaCAM increases the spreading of T24 cells on Matrigel, allowing cells to traverse the transwell chambers more easily than the cells that do not express hepaCAM.

In the cell growth control experiment, the growth rate of T24/hepaCAM was reduced about 5-fold as compared to T24/pEGFP-N2 cells (P < 0.05). The growth rate of T24/hepaCAM-mt1 cells did not differ significantly from that of T24/pEGFP-N2 cells. This result indicates that hepaCAM can inhibit the growth of T24 cells much like it does in HepG2 cells. The growth inhibitory effect that hepaCAM has on T24 cells further supports the hypothesis that hepaCAM may act a tumor suppressor in the bladder. Furthermore, when the extracellular domain of hepaCAM was deleted, the cell surface localization and the functions of hepaCAM were drastically altered. Cell-matrix adhesion, cell motility, and cell growth inhibition were notably weakened in the hepaCAM extracellular domain truncation mutants compared to wild-type hepaCAM. Taken together, these findings suggest that the extracellular domain of hepaCAM is essential to the role hepaCAM plays in cell-matrix interaction and cell motility.

## Conclusions

In conclusion, we found that hepaCAM is frequently suppressed in TCCB, the most common bladder malignancy in humans. We have also shown that hepaCAM is capable of inhibiting cell growth. These findings strongly support the hypothesis that hepaCAM may act as a tumor suppressor gene in the bladder. In addition, hepaCAM is involved in modulating cell-matrix interactions, and the extracellular domain of hepaCAM is essential to its biological and physiological functions.

## Competing interests

The authors declare that they have no competing interests.

## Authors' contributions

YFH, XHW, CLL, LW, and JL carried out the experiments described in the study. The experiments of cell related was finished by YFH, XHW, CLL, RT-PCR, clinical and patholagical features of bladder cancer were finished by LW and JL. All authors read and approved the final manuscript.

## Pre-publication history

The pre-publication history for this paper can be accessed here:

http://www.biomedcentral.com/1471-2407/10/83/prepub
